# The Patriotism of the Nurse-Training Schools

**Published:** 1917-05-12

**Authors:** 


### THE PATRIOTISM OF THE NURSE-TRAINING SCHOOLS.

### XIV.—Some Yorkshire Institutions.

It is not in our power to give a complete account of the war service rendered by Yorkshire nurses, and we feel sure that the details we are able to give will only whet the appetite for more. A list of nurses who have taken up war-work from the Sheffield Royal Hospital was given in THE HOSPITAL of November 18, 1916. It is not, however, in the largest hospitals where resources are ample that the greatest sacrifices are always to be found. The smaller establishments which have sent but two or three of their best have often been put to greater straits in proportion by dislocation of ward work than the major training-schools.

### Royal Infirmary, Huddersf eld

Early in the war a hundred beds in this infirmary were accepted by the War Office for the use of wounded soldiers, and thus all the nurses have had the privilege of sharing in the nursing of the wounded. On October 1, 1915, a military hospital containing 500 beds was opened in the town, and the following were accepted as sisters on the staff: Sisters Blackburn, Riggall, Marsh, Hawson, Inman, Brook, Rimington, and Littlewood, with staff Nurses Dodsworth and Smith. Miss Creaser, assistant matron at the Huddersfield Royal Infirmary at the outbreak of war, was appointed sister in charge of the Cambridge Hospital for wounded officers. Others nursing in connection with the war are Sister E. Fowler, hospital ship *Asturias*; Sister A. Battersby, hospital ship; Sister M. E. Walsh, military hospital, Malta; Sisters Charlesworth, Brotherton, and Beech in France; Sister Wadsworth at Cairo; Sister Leveson was among those in Serbia. Nurses Bairstow, Gartside, Wood, Brown, and Pickard, King George Hospital, London; Nurses Blakemore, Rust, Cammidge, Campbell, Fairbairn, Clayton, Huist, Fisher, Welch, Green, and Howell are working in military or Red Cross hospitals. Some of these sisters and nurses left direct from the Royal Infirmary at the beginning of the war, to take up war work; some, who had already left, rallied to the call from other hospitals, and some have since completed their training, and have gone straight to war work on receiving their certificates.

### St. Luke's (Union) Hospital, Bradford.

The history of this institution since war began forms a good illustration of the transformation wrought in Poor-Law training-schools by the necessities of the wounded. The Bowling Park Auxiliary Hospital, Bradford, was entirely staffed, with the exception of two sisters, from St. Luke's Hospital, so that there was not much room for further volunteering on the part of those trained at St. Luke's. Nevertheless, several appointments were made from the former staff. Miss Cherry, night superintendent, went to the T.F.N.S. in Liverpool; Sister Milsom to Leeds; Sisters Macnab and Dyer to the War Hospital, Epsom; and Sister Cunningham to Leicester. But within the last few months St. Luke's Hospital has been the Bradford War Hospital. Three more blocks have been built, and the beds now total 12,000. The matron and staff were retained, and of course, extra staff appointed. The civil patients were removed to Bowling Park institutions.

### Harton Hospital, South Shields.

The following is a list of the members of the staff who have been called up for duty since September 1914:—Q.A.I.M.N.S.R.—Sisters V. Bell, Palmer, Bayliff, and Coult. T.F.N.S.—Sisters Johnson, Wills, Cross, Marsh, Matthews, Fairs, Bell, Rushford, Gillesby, Cresswell, Tate, Patten, and Wilson.

The names of many other nurses trained in the hospital who are at the Front in various posts are not available.

### Scarborough Hospital and Dispensary.

The following nurses trained at this hospital are now nursing sisters under the War Office at home and abroad:—Sisters Wesolowski, Marsden, and Watson, on home service; Hewitt (France), Raine (Alexandria), Sisters Renshaw and Haseltine (Mesopotamia), Sisters Beeforth, Bayliss, and Burch (Salonica), Sister Richardson, hospital ship. Sister Cooper (trained at Leeds Infirmary) has left the Scarborough Hospital to take up war work at East Leeds. Miss Horton, trained at Derby, left the hospital for military nursing at Rouen. Sister Drake, trained at Leeds General Hospital, left Scarborough to take a post as sister at the Star and Garter, Richmond. Among those trained at Scarborough Miss Henderson is now matron of a hospital in Scotland. Many V.A.D. helpers received preparation for ward work under the matron at Scarborough.

### Stockport Infirmary.

Sister P. M. Griffith joined the Welsh Hospital at Netley early in the war, and is now a member of Q.A.I.M.N.S.R. and in Salonica. The night sister, S. Belfield, T.F.N.S., called up for service at the 2nd Western General Hospital at Manchester, returned to Stockport Infirmary a year later. Sister Ashworth, Q.A.I.M.N.S.R., is at Farnham. Nurse Poole is now in Salonica. Nurses Pownall, Whittet, Campbell, Townsend, and Young are all members of Q.A.I.M.N.S.R. Sisters Huggard and Ellison (T.F.N.S.) are at Cardiff. Many of the former Stockport nurses are in one or other of the Services, and all who finish their training pass out immediately to take up war work.

